# Neutralizing antibody responses to three XBB protein vaccines in older adults

**DOI:** 10.1038/s41392-025-02132-y

**Published:** 2025-02-03

**Authors:** Guo-Jian Yang, Mei Lu, Rui-Rui Chen, Shuang-Qing Wang, Sheng Wan, Xue-Dong Song, Guo-Ping Cao, Lei Lv, Xue-Juan He, Bing-Dong Zhan, Mai-Juan Ma

**Affiliations:** 1 Department of Microbiological Laboratory Technology, School of Public Health, Cheeloo College of Medicine, Shandong University, Jinan, 250012 China; 2 State Key Laboratory of Pathogen and Biosecurity, Academy of Military Medical Sciences, Beijing, 100071 China; 3 Kaihua Center for Disease Control and Prevention, Quzhou, 324300 China; 4 School of Public Health, Zhengzhou University, Zhengzhou, 450001 China; 5 Department of Infectious Disease Control and Prevention, Quzhou Center for Disease Control and Prevention, Quzhou, 324000 China; 6 Department of Laboratory Medicine, Handan Central Hospital, Hebei Medical University, Handan, 056001 China

**Keywords:** Infectious diseases, Molecular medicine

## Abstract

The ongoing COVID-19 pandemic has underscored the importance of strong immune defenses against emerging SARS-CoV-2 variants. While COVID-19 vaccines containing XBB subvariants have proven effective in neutralizing new SARS-CoV-2 variants, a gap remains in knowledge regarding neutralizing antibody responses in older adults aged >65 years against these newly emerged variants. This study was therefore undertaken to investigate and compare neutralizing antibody responses to three XBB-containing protein-based vaccines (trivalent XBB.1.5 vaccine, bivalent Omicron XBB vaccine, and tetravalent XBB.1 vaccine) head-to-head in 90 individuals aged >65 years. The results showed that all three XBB-containing vaccines substantially enhanced the neutralizing antibody response, with 100% of vaccinees having detectable antibody titers against ancestral D614G and variants BA.5, XBB.1.5, JN.1, KP.2, and KP.3 after booster immunization. Subsequent analysis indicated that the trivalent XBB.1.5 and tetravalent XBB.1 vaccines elicited higher levels of neutralizing antibodies compared to the bivalent Omicron XBB vaccine. The KP.2 and KP.3 variants displayed antibody resistance comparable to the JN.1 variant. Older adults produce similar neutralizing antibody responses to the vaccines regardless of their underlying medical conditions. These findings indicate that booster vaccination with XBB-containing vaccines can effectively elicit strong neutralizing responses against a number of SARS-CoV-2 variants in older adults over 65 years, which will help guide vaccine strategies in this elderly population.

## Introduction

The implementation of highly efficacious coronavirus disease 2019 (COVID-19) vaccines has mitigated the risk of infection, disease severity, hospitalization, and mortality. Nonetheless, over 4 years after the emergence of severe acute respiratory syndrome coronavirus 2 (SARS-CoV-2), the persistent evolution and spread of new variants of the virus remain to present significant global health challenges because of their increased transmissibility and resistance to vaccine-elicited neutralizing antibodies, making existing vaccines less effective in preventing infection. In this context, timely updates of COVID-19 vaccines, including those based on mRNA, vector, and protein technologies containing the SARS-CoV-2 XBB subvariants, have been developed.^1–6^ These updated XBB-containing vaccines have been demonstrated to effectively neutralize XBB lineages and the JN.1 variant,^1–6^ albeit with slightly diminished protection against the latter. Consistent with the findings of serum neutralization, the efficacy of the XBB-containing vaccines in preventing JN.1 infection was somewhat lower than their effectiveness against XBB-related lineages.^7–12^ KP.2 and KP.3, two descendants of the JN.1 variant, harbor not only recurrent spike mutations at R346T, F456L, and T572I but also have specific mutations in their spike proteins. KP.2 is characterized by mutations at positions R346T, F456L, and V1104L, while KP.3 has mutations at F456L, Q493E, and V1104L. These two subvariants have rapidly become the dominant strains in many countries as of June 2024 because of their increased transmissibility and capacity to evade immune responses.^4,13^

While neutralizing antibodies alone may not offer complete protection against SARS-CoV-2 infection, they are crucial in preventing infections and reducing the severity of the disease. Moreover, growing evidence shows that the level of neutralizing antibodies is a significant predictor of protection against infection over the first months after vaccination.^14–17^ Nonetheless, data concerning the neutralizing responses or vaccine efficacy of these XBB variant-specific vaccines in older individuals aged >65 years are lacking.^12^ Older adults face a heightened risk of severe COVID-19 complications. In contrast to earlier SARS-CoV-2 variants, the XBB lineages and the JN.1 variant cause less severe disease in adults aged <65 years, yet the risk of experiencing severe illness and death remains high in older adults aged >65 years.^18^ Moreover, older adults have a reduced efficacy in their immune response to novel antigens, a diminished capacity to produce a strong immune response after infection or vaccination,^19,20^ and a more pronounced waning of the antibody response and vaccine effectiveness.^21^ Even in the post-COVID-19 pandemic era, older adults continue to be a priority for vaccination to reduce mortality, severe illness, and hospitalizations associated with COVID-19. Most countries have prioritized vaccination for individuals older than 65 due to their increased susceptibility to infection and higher risk of severe disease.

In an effort to control infections caused by XBB subvariants, China has approved five XBB-containing COVID-19 vaccines for emergency use, including three trivalent, bivalent, and tetravalent protein-based XBB-containing vaccines. The trivalent XBB.1.5 vaccine, designed as WSK-V102C (WestVac Biopharma Co., Ltd., China), is an XBB-containing protein-based COVID-19 vaccine that utilizes the spike receptor binding domain (S-RBD) and heptad repeat motifs from the Delta, BA.5, and XBB.1.5 variants and is adjuvanted with a squalene-based oil-in-water emulsion (SE). The vaccine was authorized for emergency use for all doses or as a booster for individuals 18 years of age and older in China on June 8, 2023. The bivalent Omicron XBB vaccine, referred to as BV-01-QX (Livzon Mabpharm Inc., China), is another XBB-containing protein-based COVID-19 vaccine that utilizes S-RBD from the original Wuhan-Hu-1 strain and the Omicron XBB variant and is adjuvanted with aluminum hydroxide. The vaccine was authorized for emergency use for individuals 18 years of age and older for all doses or as a booster in China on December 1, 2023. The tetravalent XBB.1 vaccine, designated SCTV01E-2 (Sinocelltech, China), is also an XBB-containing protein-based COVID-19 vaccine based on the trimeric spike extracellular domain of the Beta, Delta, BA.1, and XBB.1 variants and is adjuvanted with SE. The vaccine was approved for emergency use for all doses or as a booster for individuals 18 years of age and older in China on December 1, 2023. Although preliminary data from manufacturers indicate that these three XBB-containing vaccines significantly boosted the level of neutralizing antibodies against earlier Omicron XBB.1.5 and EG.5.1 subvariants, the neutralizing efficacy to newly emerged variants remains to be determined.^22,23^ Moreover, there is a paucity of data regarding neutralizing responses to these three XBB-containing vaccines in older adults aged >65 years and the impact of boosters in increasing cross-reactivity against newly emerged variants.

To bridge this knowledge gap, we conducted a comparative analysis of the neutralization of D614G, BA.5, XBB.1.5, JN.1, KP.2, and KP.3 variants using serum samples from 90 volunteers aged over 65 years who received booster vaccination with the trivalent XBB.1.5 vaccine, bivalent Omicron XBB vaccine, or tetravalent XBB.1 vaccine. These data are essential not only for evaluating the neutralizing antibodies induced by these three vaccines in older adults but also for enhancing the understanding of the immune response profiles of these three vaccines, which is crucial for optimizing vaccination strategies among the elderly population.

## Results

### Study participant characteristics

A total of 90 participants over 65 years of age were included, with 30 received the trivalent XBB.1.5 vaccine, 30 received the bivalent Omicron XBB vaccine, and 30 received the tetravalent XBB.1 vaccine. The sex, age, body mass index, and smoking status distributions were comparable across all three vaccine groups (Supplementary Table 1). Approximately half of the participants reported chronic medical conditions, with hypertension being the most common condition, accounting for 59% of the reported conditions. The percentages of underlying medical conditions among the participants in the three cohorts were comparable. Eighty-eight of 90 participants completed the full course of primary vaccination, 69 of whom received their first booster vaccination, and 19 of whom received a second booster vaccination before the BA.5/BF.7 wave that occurred in late 2022 in China. All participants reported an infection or breakthrough infection during the BA.5/BF.7 wave, and more than half of them experienced an additional infection during the XBB/EG.5.1 wave in 2023. Detailed information on the study participants’ characteristics is displayed in Supplementary Table 1.

Of 90 participants vaccinated, two experienced systemic adverse effects following vaccination (Supplementary Table 1). One participant who was immunized with the trivalent XBB.1.5 vaccine experienced a general fever of 37.6 °C, and another who received the bivalent Omicron XBB vaccine experienced slight loss of appetite, fatigue, and nausea. Both participants recovered within 2–3 days after vaccination. No participant experienced serious adverse events. Detailed information on the three vaccines is summarized in Supplementary Table 2.

### Neutralizing antibodies elicited by three XBB-containing vaccines

**Fig. 1:**
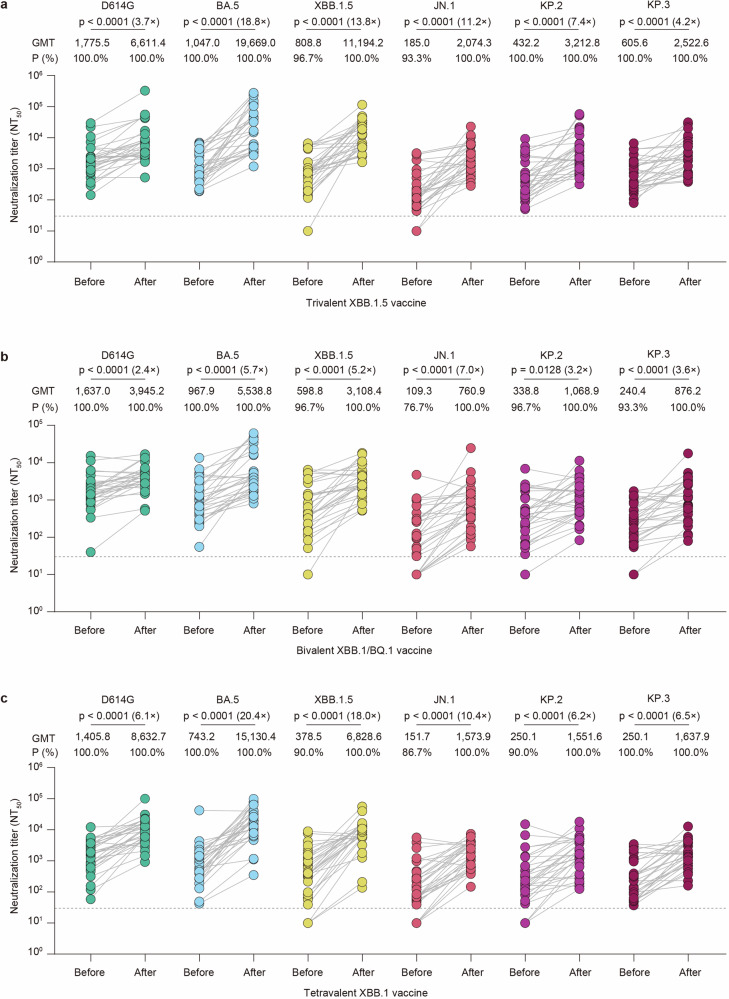
**Neutralizing antibody titers before and after immunization with three XBB-containing vaccines**. **a**–**c** Neutralization of D614G, BA.5, XBB.1.5, JN.1, KP.2, and KP.3 pseudoviruses by individual-matched serum obtained before or after vaccination with the trivalent XBB.1.5 vaccine (**a**, *n* = 30), bivalent Omicron XBB vaccine (**b**, *n* = 30), or tetravalent XBB.1 vaccine (**c**, *n* = 30). Sera were collected before vaccination (“before”) and 3 weeks after vaccination (“after”). Each dot represents the 50% neutralization titer (NT_50_) for an individual, and a line connects the NT_50_ values for the same individual before and after vaccination. The horizontal dotted line in the neutralization assay indicates a limit of detection of 30, with serum samples demonstrating neutralization below 30 plotted as 10. The geometric mean titers (GMTs) of the neutralizing antibodies and the percentages of individuals with NT_50_ values above the limit of detection are presented on top of each group. Statistical analyses were performed using a two-tailed Wilcoxon matched-pairs signed-rank test to compare neutralizing antibody titers before and after vaccination. A *p*-value <0.05 was considered statistically significant, and only significant differences are displayed in the figure, with the fold change in the GMT denoted in brackets

To assess the serum neutralizing antibody titer induced by the trivalent XBB.1.5 vaccine, bivalent Omicron XBB vaccine, and tetravalent XBB.1 vaccine, we employed a pseudovirus neutralization assay to measure neutralization titers against ancestral D614G and variants BA.5, XBB.1.5, JN.1, KP.2, and KP.2. For the trivalent XBB.1.5 vaccine, all the serum samples had neutralization titers >30 against D614G, BA.5, KP.2, and KP.3 before vaccination, whereas 96.7% (29) and 93.3% (28) of the 30 serum samples had neutralization titers >30 against XBB.1.5 and JN.1 variants, respectively (Fig. 1a). After booster immunization, all seronegative (titer < 30) serum samples were seroconverted against variants XBB.1.5 and JN.1. Compared with the GMTs before vaccination the geometric mean titers (GMTs) of D614G, BA.5, XBB.1.5, JN.1, KP.2, and KP.3 were boosted 3.7-, 18.8-, 13.8-, 11.2-, 7.4-, and 4.2-fold, respectively (Fig. 1a). For the bivalent Omicron XBB vaccine, all the serum samples had neutralization titers >30 against D614G and BA.5, whereas 96.7% (29), 76.7% (23), 96.7% (29), and 93.3% (28) of the 30 serum samples had titers >30 against the XBB.1.5, JN.1, KP.2, and KP.3 variants, respectively (Fig. 1b). After immunization, all seronegative (titer < 30) serum samples seroconverted against variants XBB.1.5, JN.1, KP.2, and KP.3. The neutralization titers against D614G, BA.5, XBB.1.5, JN.1, KP.2, and KP.3 were boosted 2.4-, 5.7-, 5.2-, 7.0-, 3.2-, and 3.6-fold, respectively, in the GMTs compared with the GMTs before vaccination (Fig. 1b). For the tetravalent XBB.1 vaccine, a similar pattern to that of the trivalent XBB.1.5 vaccine was observed for the GMTs against the tested pseudoviruses, with 6.1-, 20.4-, 18.0-, 10.4-, 6.2-, and 6.5-fold increase of the GMTs compared with the GMTs before vaccination (Fig. 1c).

**Fig. 2:**
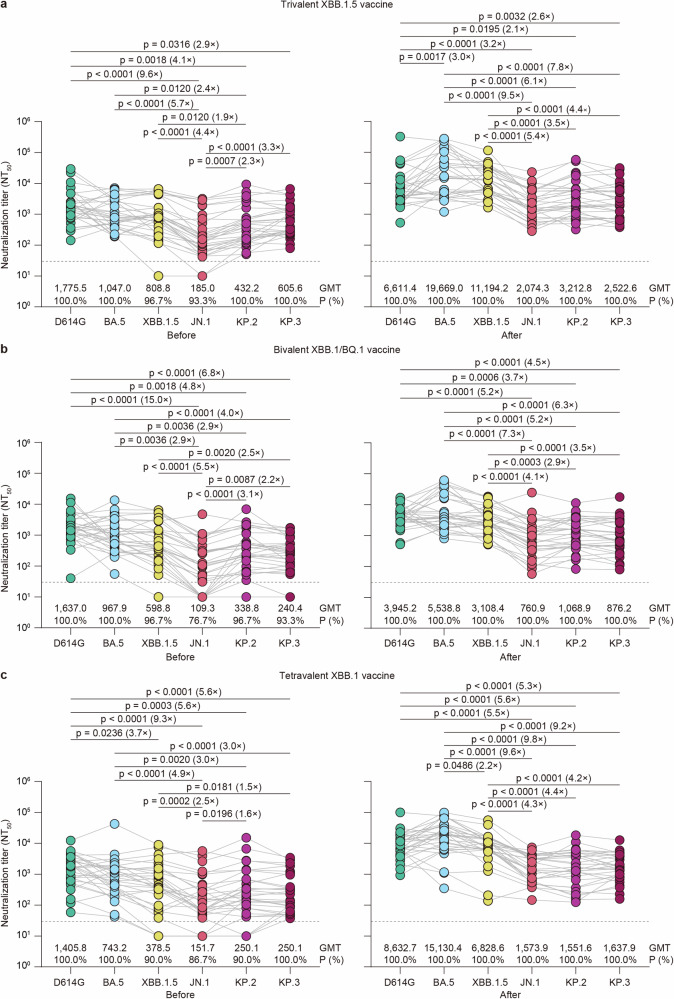
**Neutralization of the SARS-CoV-2 lineage before and after immunization with three XBB-containing vaccines**. **a**–**c** Comparison of SARS-CoV-2 lineage-specific neutralization titers against the indicated pseudoviruses before and after trivalent XBB.1.5 vaccine (**a**), bivalent Omicron XBB vaccine (**b**), and tetravalent XBB.1 vaccine (**c**) immunization. Each dot represents the 50% neutralization titer (NT_50_) for an individual. The horizontal dotted line in the neutralization assay indicates a limit of detection of 30, with serum samples demonstrating neutralization below 30 plotted as 10. The geometric mean titers (GMTs) of the neutralizing antibodies and the percentages of individuals with NT_50_ values above the limit of detection are indicated below each group. Statistical analyses were performed using a two-tailed Friedman test with a false discovery rate. A *p*-value <0.05 was considered statistically significant, and only significant differences are displayed in the figure, with the fold change in the GMT denoted in brackets

Next, we analyzed variant-specific neutralization titers stratified before and after immunization with three XBB-containing vaccines. We observed a similar neutralization pattern for the tested pseudoviruses for individuals in the three vaccine groups before vaccination. First, neutralization titers against D614G, BA.5, and XBB.1.5 were comparable (Fig. 2a–c). Second, the neutralization titer against JN.1 was the lowest. Third, KP.2 and KP.3 showed comparable neutralization escape to the JN.1 variant. After immunization, a clearer pattern was observed in which neutralizing antibody titers against JN.1, KP.2, and KP.3 variants were significantly lower than neutralization titers against the D614G, BA.5, or XBB.1.5. The neutralization titer against BA.5 in the trivalent XBB.1.5 vaccine booster group was significantly greater than the neutralizing antibody titer against D614G, and the neutralization titer against BA.5 in the tetravalent XBB.1 vaccine group was higher than the neutralization titer against XBB.1.5. Notably, there were no significant differences in neutralization titers against the JN.1, KP.2, and KP.3 variants. (Fig. 2a–c).

### Stronger antibody response induced by trivalent XBB.1.5 and tetravalent XBB.1 vaccines

**Fig. 3:**
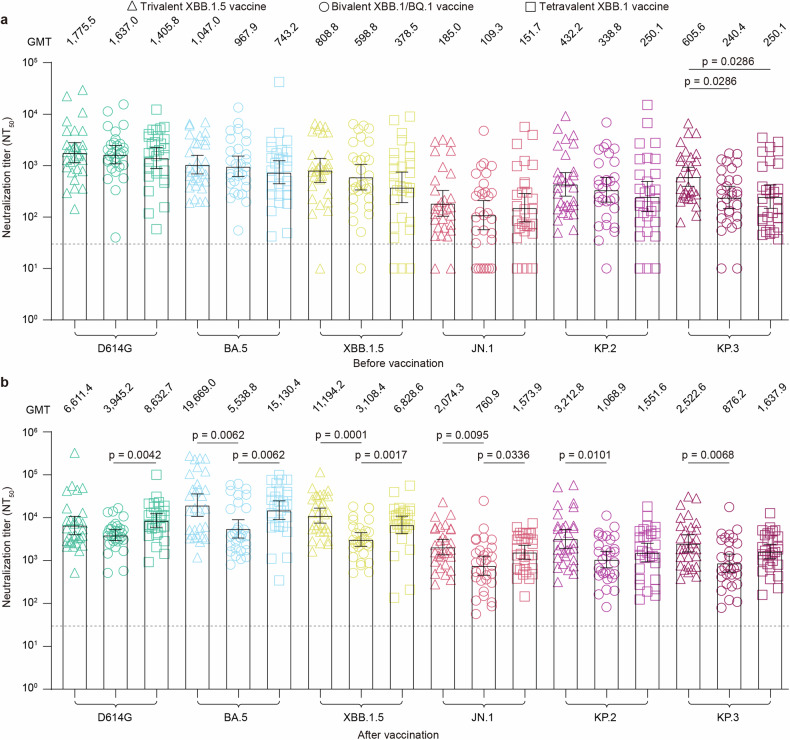
**The trivalent XBB.1.5 vaccine booster elicited increased neutralizing antibody titers**. **a**, **b** Comparison of neutralizing titers against the indicated pseudoviruses in serum collected from individuals before (**a**) and after (**b**) administration of the trivalent XBB.1.5 vaccine, bivalent Omicron XBB vaccine, or tetravalent XBB.1 vaccine. Each dot represents the 50% neutralization titer (NT_50_) for an individual. The horizontal dotted line in the neutralization assay reflects a limit of detection of 30, with serum samples exhibiting neutralization below 30 represented as 10. The geometric mean titers (GMTs) of the neutralizing antibodies are displayed on top of each group. The bar represents the GMTs and 95% confidence intervals. The dotted line indicates the limit of detection of the NT_50_. Statistical analyses were performed using the Kruskal‒Wallis test with the false discovery rate method for three-group comparisons of GMTs. A *p*-value <0.05 was considered statistically significant, and only significant differences are displayed in the figure

Next, we compared neutralization titers against the tested pseudoviruses among the three cohorts. We observed that individuals from the three cohorts before immunization had comparable neutralization titers against D614G, BA.5, XBB.1.5, JN.1, and KP.2 (Fig. 3a). However, individuals from the trivalent XBB.1.5 cohort before immunization had even higher neutralizing antibody titers against KP.3 (Fig. 3a). After vaccination, individuals in the trivalent XBB.1.5 vaccine and tetravalent XBB.1 vaccine cohorts exhibited increased neutralization titers against BA.5, XBB.1.5, and JN.1 (Fig. 3b). In contrast, individuals from the trivalent XBB.1.5 cohort produced higher neutralizing antibody titers against the variants KP.2 and KP.3, and individuals from the tetravalent XBB.1 vaccine cohort produced higher neutralizing antibody titers against D614G (Fig. 3b). Overall, recipients of trivalent XBB.1.5 and tetravalent XBB.1 likely produced similar antibody titers against the indicated pseudoviruses but were significantly higher than those of bivalent Omicron XBB vaccine recipients.

### Neutralizing antibody titers in individuals with underlying medical conditions

**Fig. 4:**
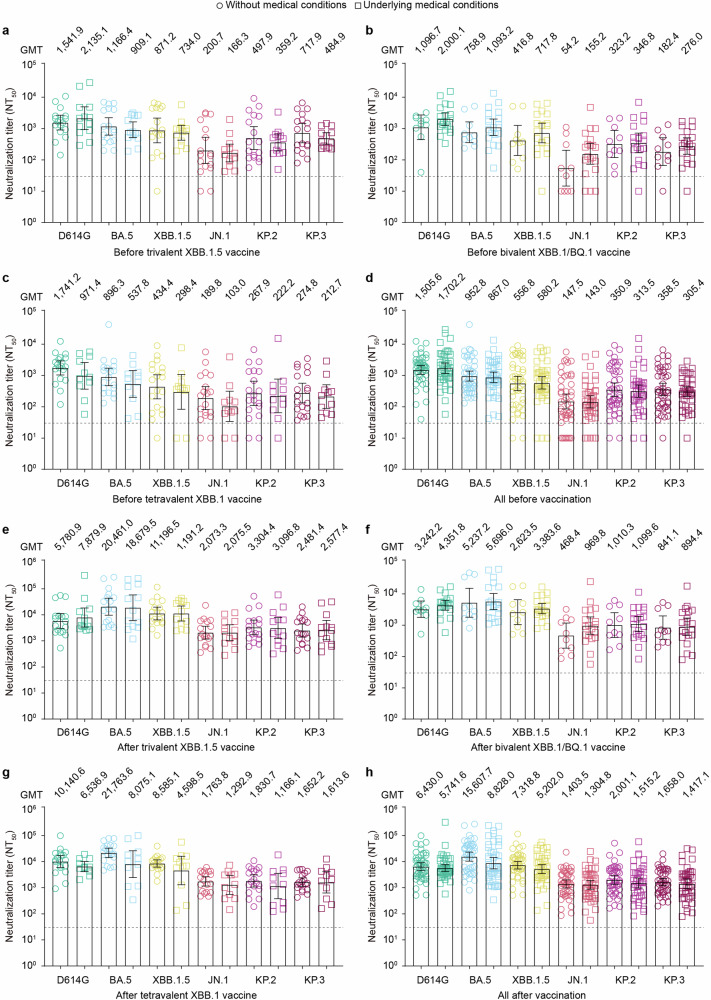
**Neutralizing antibody responses in individuals with or without medical conditions before and after immunization with three XBB-containing vaccines**. **a**–**d** Comparison of the 50% neutralization titer (NT_50_) against the indicated pseudoviruses in individuals with (*n* = 13) and without (*n* = 17) medical conditions before receiving the trivalent XBB.1.5 booster (**a**), in individuals with (*n* = 20) and without (*n* = 10) medical conditions before receiving the bivalent Omicron XBB vaccine (**b**), in individuals with (*n* = 11) and without (*n* = 19) medical conditions before receiving the tetravalent XBB.1 vaccine (**c**), and in all pooled sera (**d**). **e**–**h** NT_50_ of individuals with and without medical conditions after immunization with the trivalent XBB.1.5 booster (**e**), bivalent Omicron XBB vaccine (**f**), tetravalent XBB.1 vaccine (**g**), or all pooled sera after immunization (**h**). Each dot represents the NT_50_ for an individual. The horizontal dotted line in the neutralization assay reflects a limit of detection of 30, with serum samples exhibiting neutralization below 30 represented as 10. The bar represents the GMTs and 95% confidence intervals. Statistical analyses were performed using the Wilcoxon rank-sum test for group comparisons of GMTs between individuals with and without chronic conditions. A *p*-value <0.05 was considered statistically significant, and only significant differences are displayed in the figure

Considering that half of the participants reported underlying medical conditions, we compared antibody titers between participants with and without underlying medical conditions. The results showed that antibody titers against the tested pseudoviruses in individuals without chronic medical conditions were similar to those in individuals with chronic medical conditions, both before and after immunization (Fig. 4a, b). A further sub-analysis comparing neutralization titers between participants with hypertension and those without medical conditions revealed no statistically significant differences (Supplementary Fig. 1).

### Antigenic cartography

**Fig. 5:**
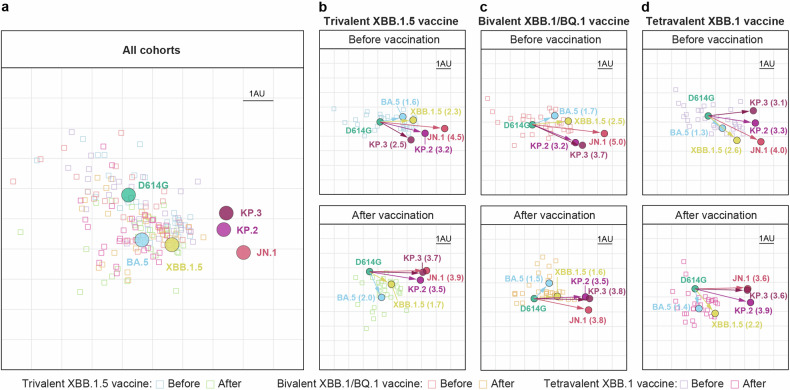
**Antigenic map of serum virus neutralization data**. **a**–**d** Antigenic maps were constructed using the Racmacs program (1.1.4) for neutralization titers against the indicated pseudoviruses from all cohorts (**a**), individuals before and after receiving the trivalent XBB.1.5 vaccine (**b**), bivalent Omicron XBB vaccine (**c**), and tetravalent XBB.1 vaccine (**d**). The circles represent the indicated variants, whereas the squares denote individual serum samples. The *x*- and *y*-axes depict antigenic units (AUs), with each grid corresponding to a 2-fold serum dilution of the neutralization titer. One square on the grid corresponds to one AU squared. Arrows between D614G and selected variants are annotated with the distance between those variants in AUs

By employing pooled and separate serum neutralization data from all three vaccine cohorts, antigenic maps were created to quantify and illustrate the antigenic disparities between ancestral D614G and the tested variants (Fig. 5a–d). The map shows that the sera from the three cohorts before vaccination substantially overlapped and was centered around D614G, and the sera from the three cohorts after immunization shifted toward the BA.5 and XBB.1.5 variants (Fig. 5a). The JN.1, KP.2, and KP.3 variants were clustered together, showing greater antigenic distinction from D614G than from the XBB.1.5 variant (Fig. 5a). Specifically, the antigenic distances between D614G and the XBB.1.5 and JN.1 variants after the administration of the three XBB-containing vaccines indicated a significant boost in antibody potency and breadth (Fig. 5b, c). However, the antigenic distances between D614G and KP.2 or KP.3 were not shorter after immunization than before vaccination (Fig. 5b, c), suggesting that XBB-containing vaccines boost the antibody potency and breadth to KP.2 and KP.3 but are limited, which is consistent with a mean 5- and 4-fold increase in GMTs being also observed after immunization (Fig. 1). Taken together, the JN.1, KP.2, and KP.3 variants exhibited similar antigenic and distant properties.

## Discussion

Vaccination with COVID-19 XBB-containing vaccines has significantly boosted the neutralizing antibody response to XBB lineages, the JN.1 variant and its subvariants KP.2 and KP.3, and protection against XBB lineages or JN.1 infection.^24^ However, neutralizing antibody immune responses to XBB-containing vaccine immunization in older adults, as well as their cross-reactivity with variants of concern in older adults, are lacking. Here, we quantified the levels of neutralizing antibodies elicited by the bivalent Omicron XBB vaccine, the trivalent XBB.1.5 vaccine, and the tetravalent XBB.1 vaccine, highlighting their implications for understanding the neutralizing antibody response associated with each vaccine. A key strength of our study is that blood samples after the vaccination with three XBB-containing vaccines were collected at a consistent time between the administration of the vaccine and the subsequent blood sampling process. Additionally, all the samples were processed at a single blood processing facility and analyzed simultaneously using unified experimental technologies.

In the current study, neutralizing antibodies were detected in all participants after immunization. Neutralizing antibody titers against ancestral D614G and variants XBB.1.5, JN.1, KP.2, and KP.3 were significantly boosted. Notably, there was a more substantial increase in the GMTs of the neutralization titer against the XBB.1.5 and JN.1 variants, which is in line with previous studies conducted in younger adults.^2,22^ At 21 days after vaccination, the hierarchy of neutralizing antibody titers among the three vaccines was trivalent XBB.1.5 vaccine ≈ tetravalent XBB.1 vaccine > bivalent Omicron XBB vaccine. This difference may be attributed to the different spike proteins contained in the three vaccines, such as XBB.1.5, BA.5, and Delta spike in the trivalent XBB.1.5 vaccine; ancestral Wuhan-Hu-1 and Omicron XBB in the bivalent Omicron XBB vaccine; and Beta, BA.1, BQ.1.1, and XBB.1 in the tetravalent XBB.1 vaccine. However, whether immune imprinting involves a relatively weak antibody response to the bivalent Omicron XBB vaccine should be further investigated, as previous studies have reported immunological imprinting after repeated ancestral SARS-CoV-2 antigen exposure.^25–30^ On the other hand, one recent study by Liang et al. revealed that individuals who received two Omicron-matched booster doses after the original mRNA-1273 vaccine could bind to the Omicron spike protein and neutralize related sarbecoviruses.^31^ However, neutralizing responses were diminished when the serum was pre-cleared with the Wuhan-Hu-1 spike protein. This finding indicates that earlier mRNA-1273 vaccinations promote the induction of cross-neutralizing antibodies, which help target emerging SARS-CoV-2 variants and related viruses, offering broader protection.

We also found that antibody responses in older adults with medical conditions were comparable to those in older adults without underlying medical conditions. Our findings are consistent with several studies that hypertension and diabetes are not correlated with a diminished antibody response following COVID-19 vaccination^32–34^ but are inconsistent with other studies that individuals with hypertension and diabetes exhibit lower levels of spike-specific IgG antibodies after immunization with the COVID-19 vaccine.^35–38^ A recent study reported that individuals with untreated hypertension, as well as those with diabetes, regardless of whether they were untreated or treated, exhibited lower levels of spike-specific IgG antibody titers compared to individuals without these medical conditions.^39^ Similar controversial results have also been reported regarding cardiovascular disease, chronic lung disease, and cancer.^36,39–41^ In light of the inconsistent findings from our study and those from previous studies, it is necessary to continuously monitor antibody titers in individuals with medical conditions, such as hypertension or diabetes, and additional booster shots may also be needed to sustain immunity.

Since the most recent immunogenicity studies have focused on the most widely used COVID-19 mRNA vaccines, immunogenicity studies on non-mRNA vaccines are crucial. Reassuringly, three protein-based XBB-containing vaccine boosters elicit strong neutralizing antibody responses in older adults over 65 years of age. Nonetheless, further investigations are needed to assess the effects of age on the persistence of immunity after booster immunization and their efficacy against new variants. While the decline in immunity against SARS-CoV-2 in older adults may be quicker than that in younger adults, booster immunization with XBB-containing vaccines can induce broadly neutralizing antibodies against SARS-CoV-2 variants in this high-risk population. Additionally, protein-based vaccines are widely recommended for administration in infants, children, adults, and even in elderly individuals, and their promising safety profile and benefits are well-recognized. In addition, protein-based vaccines are expected to reach the clinic faster than nucleic acid-based or vector-based vaccines.^42^ Collectively, these findings underscore the recommendation for the deployment of these vaccines in older adults to improve immunity against current and emerging SARS-CoV-2 variants.

Several limitations should be addressed in this study. First, the relatively small sample size may limit the generalizability of the study findings. In addition, the lack of a younger reference group may also limit our understanding of age-related differences in antibody responses to XBB-containing vaccines. Furthermore, the data on medical conditions and infection histories may be inaccurate because of the recall bias collected through questionnaires, which can lead to misrecording health-related claims or infection times. Second, neutralizing responses elicited by alternative vaccine platforms, including mRNA-based and vector-based vaccines, were not assessed because the vaccines were limited in utilization in China or unavailable at the study site. Moreover, given that systemically administered vaccines induce limited mucosal immune responses,^43,44^ our study focused on the systemic immune response. Therefore, mucosal IgA antibodies, which are also critical for preventing severe outcomes from SARS-CoV-2 infection,^45^ were not evaluated. Third, the study did not assess the T-cell response, which is also essential for protective immunity against infection. Fourth, antibody responses were measured ~1 month after vaccination. Longitudinal studies of neutralizing antibodies following booster vaccination are needed to elevate the durability of immunity induced by the three XBB-containing vaccines against new variants. Lastly, the increasing complexity of population heterogeneity after breakthrough infections and vaccinations has led to significant difficulties in recruiting participants with similar backgrounds regarding infection and vaccination status. As a result, this heterogeneity among the study participants may influence antibody responses after immunization with XBB-containing vaccines.

In summary, our study presents important evidence of neutralizing antibody responses induced by three protein-based XBB-containing vaccines in older adults aged over 65 years. This study also provides significant insights for optimizing vaccination strategies for older adults to enhance neutralizing immunity against emerging SARS-CoV-2 variants. On the other hand, investigations are needed to characterize the immunity elicited by vaccines with different routes of administration.

## Material and methods

### Study design, participants, and sampling

In April 2024, older adults aged >65 years who were planning to receive XBB-containing vaccines were recruited from a local community service center in Quzhou City, Zhejiang Province, to compare neutralizing antibody responses to three XBB-containing recombinant protein vaccines. These three XBB-containing vaccines include a trivalent XBB.1.5 protein vaccine (WSK-V102C; WestVac Biopharma Co., Ltd., China), a bivalent XBB protein vaccine (BV-01-QX; Livzon Mabpharm Inc., China), and a tetravalent XBB.1 protein vaccine (SCTV01E-2; Sinocelltech, China). Owing to the decreased demand for COVID-19 vaccines, each vaccine has 30 doses available for immunization. Therefore, 30 individuals were enrolled for each vaccine and included in the study. The sample size for this study was not pre-determined using statistical methods. Instead, it was chosen to align with the sample sizes of previous studies for evaluating neutralizing antibody response following SARS-CoV-2 infection and vaccination,^46–48^ as well as the available vaccine dose at the study site.

Individuals who met the following criteria were included in the study: (1) were older than 65 years, (2) were healthy or had stable, well-controlled chronic conditions, and (3) had completed the primary immunization series, received booster doses, or had a SARS-CoV-2 infection for >6 months. Conversely, individuals were excluded if they had a history of immunosuppressive medications, antibacterial agents, corticosteroids, immunosuppressive drugs, anesthetic agents, severe allergic reactions to vaccine components, or an infection of SARS-CoV-2 within the preceding 14 days.

We employed the RUND function in Microsoft Excel to generate a sequence of random numbers ranging from 001–090. Each random number was divided by 30 to create a fixed random identifier, determining the participants’ allocation to one of the three vaccine groups. Participants with random numbers less than or equal to 1, those between 1 and 2 were assigned vaccine 2, and participants with numbers >2 were assigned vaccine 3. The participants were then given a permanent study number based on the order of their arrival at the study site. These study numbers were then matched with the generated random identifiers to allocate each participant to their respective vaccine group. After vaccination, the participants were asked daily for 7 days to report whether they experienced systemic adverse effects. Systemic adverse effects included fever, headache, fatigue, chills and shivers, diarrhea, arthralgia, myalgia, and nausea.

We collected serum samples from all participants who were subsequently vaccinated with these three XBB-containing vaccines on the same day, and we conducted a follow-up evaluation 21 days later. We collected venous blood samples from the participants, centrifuged them to obtain plasma containing neutralizing antibodies, and then detected and calculated the antibody content in the plasma through pseudovirus neutralization tests. Additionally, we collected demographic information and information related to exposure, such as vaccination history and infection history, through online forms and paper-based questionnaires. The vaccination records of each participant were verified through the vaccination system.

This study was conducted in accordance with the Declaration of Helsinki and approved by the Institutional Review Board of the Academy of Military Medical Sciences (AF/SC-08/02.197). Each participant provided informed written consent.

### Cell lines

HEK-293T cells (ATCC, CRL-3216) were propagated at 37°C with 5% CO_2_ in Dulbecco’s Modified Eagle’s Medium (DMEM, Gibco), supplemented with 10% (v/v) heat-inactivated fetal bovine serum (FBS, Gibco) and 1% penicillin-streptomycin (Gibco). The cells were passaged when they reached confluence, using 0.25% trypsin with 1 mM EDTA (Solarbio) every 48–72 h. HEK-293T cells expressing human ACE2 (HEK-293T-hACE2) were propagated under identical conditions.

### Spike plasmid pseudovirus production

A pseudoviruses were produced by cotransfecting HEK-293T cells with human immunodeficiency virus backbones expressing firefly luciferase (pNL4-3-R-E-luciferase) and the pcDNA3.1 vector encoding the spike proteins of the D614G, XBB.1.5, JN.1, KP.2, and KP.3 plasmids. These codon-optimized, full-length open reading frames were synthesized by GenScript (Nanjing, China). All the plasmid-encoded spike sequences were confirmed by Sanger sequencing. The mutations in the spike proteins of D614G, BA.5, XBB.1.5, and JN.1 were described in our previous studies,^49,50^ while the mutations in the spike proteins of KP.2 and KP.3 relative to D614G are shown in Supplementary Table 3. Pseudovirus particles were generated by cotransfecting HEK-293T cells with human immunodeficiency virus backbones expressing firefly luciferase (pNL4-3-R-E-luciferase) and the pcDNA3.1 vector encoding the spike proteins D614G, XBB.1.5, JN.1, KP.2, and KP.3 plasmids. The culture medium was replaced with fresh medium at 24 h, and the supernatants were harvested at 48 h posttransfection and clarified by centrifugation at 300 × *g* for 10 min before being aliquoted and stored at -80 °C until use.

### Pseudovirus neutralization assay

Pseudovirus neutralization assay (pVNT) was performed as previously described,^49,50^ utilizing the 293 T cell line stably expressing human ACE2 orthologs as target cells. All viruses were first titrated to normalize the viral input across assays. Duplicate 3-fold serial 8-point dilutions of heat-inactivated sera, starting at a 1:30 dilution, were mixed with 500-1000 TCID_50_ of the SARS-CoV-2 pseudotyped virus and incubated for 1 h at 37 °C and 5% CO_2_. Subsequently, 1 × 10^4^ 293T-ACE2 cells were added per well and incubated for 48 h at 37°C and 5% CO_2_. Afterward, the supernatant was removed, and the cells were lysed with a passive lysis buffer (Vazyme) for 3 min at room temperature. The lysates were transferred to opaque white 96-well plates, mixed with luciferase assay buffer (Vazyme, China), and luminescence was measured immediately using a GloMax 96 Microplate Luminometer (Promega). The 50% neutralization titer (NT_50_) was calculated using a four-parameter nonlinear regression inhibitor curve in GraphPad Prism 9.0.0 (GraphPad Software), with the NT_50_ defined as the reciprocal serum dilution that elicits a 50% reduction in relative light units. Samples with an NT_50_ value below 30 (the detection threshold) were classified as negative for neutralizing antibodies and were assigned a nominal value of 10 for geometric mean titer calculations, which is the lowest serum dilution factor used in the pseudovirus neutralization assay.

### Antigenic cartography

An antigenic map was constructed employing a previously outlined antigenic cartography approach. The antigenic distances among SARS-CoV-2 variants were estimated by incorporating the neutralization efficacy of each serum sample, with these distances inversely proportional to the log2 titer of the antigens and antisera. The Racmacs package (https://acorg.github.io/Racmacs/, v.1.1.4) within R was utilized to generate the map, employing 2000 optimization iterations and setting the minimum column basis parameter to “none”. The map distances function of the Racmacs package was used to determine antigenic distances, and the average distances for all sera to variants were utilized to represent the final distances. Within each serum group, D614G served as the reference point for the serum samples, and the seeds for each antigenic map were manually adjusted to ensure that XBB.1.5 was displayed horizontally relative to the serum samples.

### Statistical analysis

A descriptive analysis was employed to summarize the demographic characteristics of the participants, presenting relative counts and frequencies for categorical variables, as well as medians with interquartile ranges for nonnormally distributed continuous variables. The chi-square test or Fisher’s exact test was used to compare the categorical variables among different cohorts as appropriate. The Wilcoxon matched-pairs signed rank test was applied to compare the neutralizing antibody titers in paired samples collected before and after vaccination. The Friedman test with the false discovery rate method was used for multiple comparisons of the neutralizing antibody titers among paired neutralizing antibody titers against the tested pseudoviruses of participants. The Kruskal‒Wallis test with the false discovery rate method was applied for multiple comparisons of the ages, unpaired neutralizing antibody titers, and interval days between the last vaccination or infection and sampling. Statistical analyses were conducted utilizing GraphPad Prism software (version 9.0.0, La Jolla, California, USA). All statistical tests were 2-sided with a significance level of 0.05. Further information regarding the statistical analysis is provided in the figure legends.

## Supplementary information

Supplementary Materials

## Data Availability

All data that support the results of this study are included in the main text and supplementary information. Raw data and further information are available from the corresponding author upon reasonable request.
