# Supplementary material for: Neutralizing antibody responses to three XBB protein vaccines in older adults
Source: Signal Transduct Target Ther. 2025 Feb 3;10:48. doi: 10.1038/s41392-025-02132-y (PMC11788433; doi:10.1038/s41392-025-02132-y)
Supplement: Supplementary file 1 — Supplementary Materials [file 41392_2025_2132_MOESM1_ESM.docx]

Supplementary Materials for

Neutralizing antibody responses to three XBB protein vaccines in older adults

Guo-Jian Yang, Mei Lu, Rui-Rui Chen, Shuang-Qing Wang, Sheng Wan, Xue-Dong Song, Guo-Ping Cao, Lei Lv, Xue-Juan He, Bing-Dong Zhan, Mai-Juan Ma

Correspondence to: bd_zhan@126.com; mjma@163.com

**This PDF file includes:**

Figures. S1

Tables S1 to S3


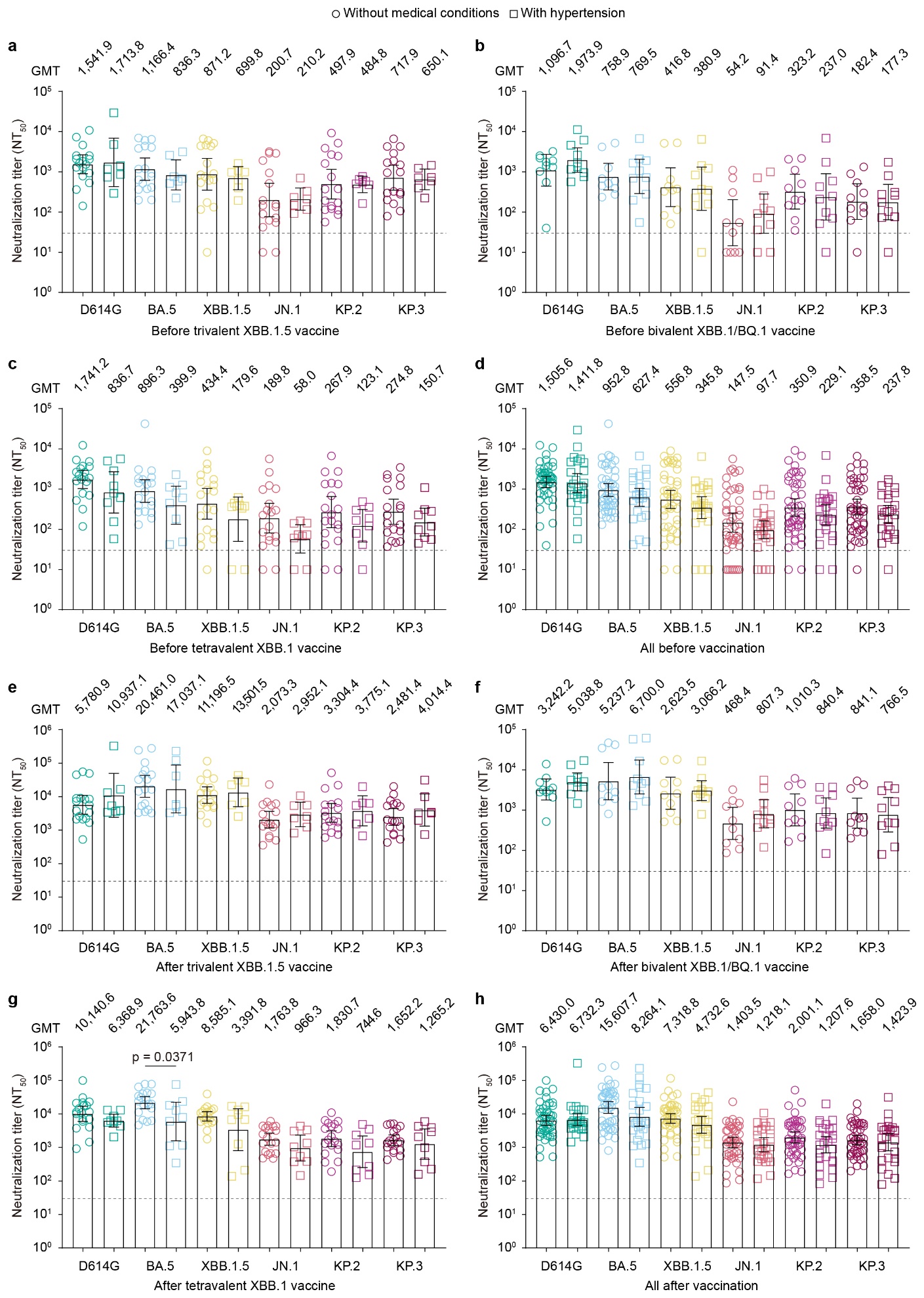


Figure. S1. Neutralizing antibody responses in individuals with hypertension or without underlying medical conditions before and after immunization with three XBB-containing vaccines.

(**a-d**) Comparison of the 50% neutralization titer (NT50) in individuals with (n =7) hypertension and without (n = 17) medical conditions before receiving the trivalent XBB.1.5 booster (**a**), in individuals with (n =10) hypertension and without (n = 10) medical conditions before receiving the bivalent Omicron XBB vaccine (**b**), in individuals with (n = 9) hypertension and without (n =19) medical conditions before receiving the tetravalent XBB.1 vaccine (**c**), and in all pooled sera (**d**). (**e-h**) NT50 of individuals with and without medical conditions after immunization with the trivalent XBB.1.5 booster (**e**), bivalent Omicron XBB vaccine (**f**), tetravalent XBB.1 vaccine (**g**), or all pooled sera after immunization (**h**). Each dot represents the NT50 for an individual. The horizontal dotted line in the neutralization assay reflects a limit of detection of 30, with serum samples exhibiting neutralization below 30 represented as 10. The bar represents geometric mean titers and 95% confidence intervals. Statistical analyses were performed using the Wilcoxon rank-sum test for group comparisons between individuals with hypertension and without underlying medical conditions.

Table S1. Demographic characteristics of the study participants.

| **Characteristics** | **Trivalent XBB.1.5 vaccine** | **Bivalent XBB.1/BQ.1 vaccine** | **Tetravalent XBB.1 vaccine** | ***p*-value** |
| --- | --- | --- | --- | --- |
| **No. of participants** | 30 | 30 | 30 | 1.00 |
| **Age (median, IQR)** | 70.0 (68.0-73.0) | 68.5 (66.0-72.0) | 69.0 (66.8-72.0) | 0.07 |
| **Sex (n, %)** |  |  |  | 0.39 |
| Male | 18 (60.0) | 14 (46.7) | 19 (63.3) |  |
| Female | 12 (40.0) | 16 (53.3) | 11 (36.7) |  |
| **Body mass index (median, IQR)** | 22.4 (19.5-25.8) | 23.3 (19.5-25.1) | 23.9 (21.2-25.7) | 0.70 |
| **Smoking status (n, %)** |  |  |  | 0.18 |
| Smokers | 8 (26.7) | 6 (20.0) | 13 (43.3) |  |
| Never smokers | 22 (73.3) | 23 (76.7) | 16 (53.3) |  |
| Ex-smokers | 0 | 1 (3.3) | 1 (3.3) |  |
| **Underlying Medical conditions (n, %)** |  |  |  | 0.06 |
| Hypertension | 7 (23.3) | 10 (33.3) | 9 (30.0) |  |
| Chronic obstructive pulmonary disease | 3 (10.0) | 3 (10.0) | 0 |  |
| Diabetes | 3 (10.0) | 2 (6.7) | 0 |  |
| Heart disease | 0 | 4 (13.3) | 2 (6.7) |  |
| Hyperuricemia | 0 | 1 (3.3) | 0 |  |
| No | 17 (56.7) | 10 (33.3) | 19 (63.3) |  |
| **Unvaccinated participants (n, %)** | 0 | 2 (6.7) | 0 | 0.33 |
| **Primary Vaccination (n, %)** |  |  |  |  |
| 2🞨CoronaVac/BBIBP-CorV | 30 (100.0.0) | 28 (93.3) | 30 (100.0) | 0.33 |
| **One booster before breakthrough (n, %)** |  |  |  | 0.01 |
| 1🞨ZF2001 | 0 | 0 | 5 (16.7) |  |
| 1🞨Ad5-nCoV* | 0 | 0 | 1 (3.3) |  |
| 1🞨CoronaVac/BBIBP-CorV | 21 (70.0) | 21 (70.0) | 21 (70.0) |  |
| **Two boosters before breakthrough (n, %)** |  |  |  | 0.16 |
| 1🞨CoronaVac/BBIBP-CorV + 1🞨ZF2001 | 5 (16.7) | 2 (6.7) | 0 |  |
| 1🞨CoronaVac + 1🞨Ad5-nCoV** | 4 (13.3) | 5 (16.7) | 2 (6.7) |  |
| 1🞨ZF2001 + 1🞨ZF2001 | 0 | 0 | 1 (3.3) |  |
| **Sample follow-up sampling time** |  |  |  |  |
| Days post-vaccination (median, IQR) | 21.0 (21.0-21.0) | 21.0 (21.0-21.0) | 21.0 (21.0-21.0) | 1.00 |
| **Systemic adverse effects (n, %)** |  |  |  | NA |
| Fever | 1 (3.3) | 0 | 0 |  |
| Loss of appetite | 0 | 1 (3.3) | 0 |  |
| Fatigue | 0 | 1 (3.3) | 0 |  |
| Nausea | 0 | 1 (3.3) | 0 |  |

IQR, interquartile range. CoronaVac, Sinovac, China. BBIBP-CorV, Sinopharm, China. ZF2001, Anhui Zhifei Longcom, China. *Intramuscular Ad5-nCoV, CanSinoBIO, China. **Aerosolized Ad5-nCoV, CanSinoBIO, China. NA, not available.

Table S2. Information of six COVID-19 vaccines related in this study.

| **Vaccine** | **CoronaVac** | **BBIBP-CorV** | **ZF2001** | **WSK-V102C** | **BV-01-QX** | **SCTV01E-2** |
| --- | --- | --- | --- | --- | --- | --- |
| **Vaccine platform** | Inactivated | Inactivated | Protein subunit | Protein subunit | Protein subunit | Protein subunit |
| **Type of antigen** | Whole virus | Whole virus | RBD | RBD | RBD | Spike |
| **Viral strain** | Wuhan | Wuhan | Wuhan | XBB.1.5+BA.5+Delta | Wuhan+Omicron XBB | Beta+BA.1+BQ.1.1+XBB.1 |
| **Adjuvant type** | AH | AH | AH | SE | AH | SE |
| **Dosage** | 3 μg/0.5 ml | 4 μg/0.5 ml | 25 μg/0.5 ml | 30 μg/0.25 ml | 20 μg/0.5 ml | 30 μg/0.5 ml |
| **Number of doses** | 2 | 2 | 2 or 3 | 3 | NA | 2 |
| **Schedule** |  |  |  |  |  |  |
| Primary (day) | 0+14 | 0+21 | 0+28 or 0+28+56 | 0+21+42 | NA | 0+28 |
| Booster* | ≥ 6 months | ≥ 6 months | ≥ 6 months | ≥ 6 months | ≥ 6 months | ≥ 6 months |
| **Route of administration** | IM | IM | IM | IM | IM | IM |
| **Manufacturer** | Sinovac, China | Sinopharm, China | AnhuiZhifei Longcom, China | WestVac Biopharma Co., Ltd., China | Livzon Mabpharm Inc. China | Sinocelltech, China |

AH, aluminum hydroxide; SE, squalene-based oil-in-water emulsion; IM, intramuscular injection; RBD, receptor binding domain. NA, not available. *Vaccination since last vaccination or infection.

Table S3. Mutations in the spike protein of variants used in the study.

| **Variants** | **Mutations** |
| --- | --- |
| **KP.2** | ins16_ MPLF, T19I, R21T, Δ24-26, A27S, S50L, Δ69-70, V127F, G142D, Δ144, F157S, R158G, Δ211, L212I, V213G, L216F, H245N, A246D, I332V, G339H, R346T, K356T, S371F, S373P, S375F, T376A, R403K, D405N, R408S, K417N, N440K, V445H, G446S, N450D, L452W, L455S, F456L, N460K, S477N, T478K, N481K, Δ483, E484K, F486P, Q498R, N501Y, Y505H, E554K, A570V, D614G, P621S, H655Y, N679K, P681R, N679K, N764K, D796Y, S939F, Q954H, N969K, V1104L, P1143L |
| **KP.3** | ins16_ MPLF, T19I, R21T, Δ24-26, A27S, S50L, Δ69-70, V127F, G142D, Δ144, F157S, R158G, Δ211, L212I, V213G, L216F, H245N, A246D, I332V, G339H, K356T, S371F, S373P, S375F, T376A, R403K, D405N, R408S, K417N, N440K, V445H, G446S, N450D, L452W, L455S, F456L, N460K, S477N, T478K, N481K, Δ483, E484K, F486P, Q493E, Q498R, N501Y, Y505H, E554K, A570V, D614G, P621S, H655Y, N679K, P681R, N679K, N764K, D796Y, S939F, Q954H, N969K, V1104L, P1143L |
